# Novel effects of the gastrointestinal hormone secretin on cardiac metabolism and renal function

**DOI:** 10.1152/ajpendo.00260.2021

**Published:** 2021-11-22

**Authors:** Sanna Laurila, Eleni Rebelos, Minna Lahesmaa, Lihua Sun, Katharina Schnabl, Tia-Mari Peltomaa, Riku Klén, Mueez U-Din, Miikka-Juhani Honka, Olli Eskola, Anna K. Kirjavainen, Lauri Nummenmaa, Martin Klingenspor, Kirsi A. Virtanen, Pirjo Nuutila

**Affiliations:** ^1^Turku PET Centre, University of Turku, Turku, Finland; ^2^Heart Center, Turku University Hospital, Turku, Finland; ^3^Heart Center, Satakunta Central Hospital, Pori, Finland; ^4^Department of Internal Medicine, Jorvi Hospital, Helsinki University Hospital, Helsinki, Finland; ^5^Chair for Molecular Nutritional Medicine, TUM School of Life Sciences, Technical University of Munich, Freising, Germany; ^6^EKFZ - Else Kröner Fresenius Center for Nutritional Medicine, Technical University of Munich, Freising, Germany; ^7^ZIEL – Institute for Food & Health, Technical University of Munich, Freising, Germany; ^8^Turku PET Centre, Turku University Hospital, Turku, Finland; ^9^Department of Psychology, University of Turku, Turku, Finland; ^10^Institute of Public Health and Clinical Nutrition, University of Eastern Finland (UEF), Kuopio, Finland; ^11^Department of Endocrinology and Clinical Nutrition, Kuopio University Hospital, Kuopio, Finland; ^12^Department of Endocrinology, Turku University Hospital, Turku, Finland

**Keywords:** gastrointestinal hormone, kidney function, myocardial metabolism, secretin

## Abstract

The cardiac benefits of gastrointestinal hormones have been of interest in recent years. The aim of this study was to explore the myocardial and renal effects of the gastrointestinal hormone secretin in the GUTBAT trial (NCT03290846). A placebo-controlled crossover study was conducted on 15 healthy males in fasting conditions, where subjects were blinded to the intervention. Myocardial glucose uptake was measured with [^18^F]2-fluoro-2-deoxy-d-glucose ([^18^F]FDG) positron emission tomography. Kidney function was measured with [^18^F]FDG renal clearance and estimated glomerular filtration rate (eGFR). Secretin increased myocardial glucose uptake compared with placebo (secretin vs. placebo, means ± SD, 15.5 ± 7.4 vs. 9.7 ± 4.9 μmol/100 g/min, 95% confidence interval (CI) [2.2, 9.4], *P* = 0.004). Secretin also increased [^18^F]FDG renal clearance (44.5 ± 5.4 vs. 39.5 ± 8.5 mL/min, 95%CI [1.9, 8.1], *P* = 0.004), and eGFR was significantly increased from baseline after secretin, compared with placebo (17.8 ± 9.8 vs. 6.0 ± 5.2 ΔmL/min/1.73 m^2^, 95%CI [6.0, 17.6], *P* = 0.001). Our results implicate that secretin increases heart work and renal filtration, making it an interesting drug candidate for future studies in heart and kidney failure.

**NEW & NOTEWORTHY** Secretin increases myocardial glucose uptake compared with placebo, supporting a previously proposed inotropic effect. Secretin also increased renal filtration rate.

## Graphical abstract

**Figure:**
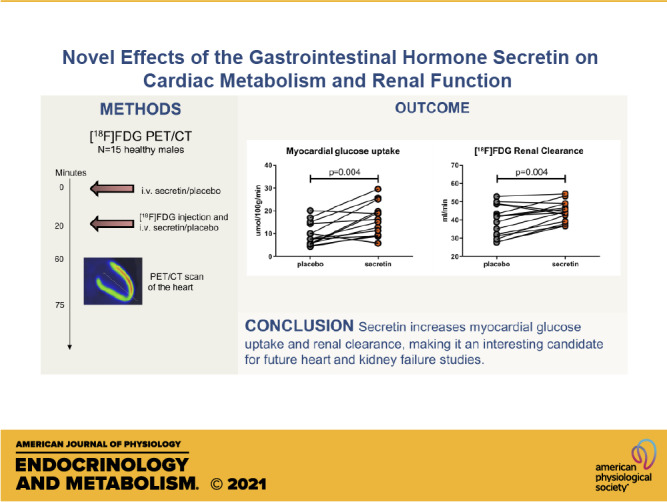

## INTRODUCTION

The cardiac benefits of gastrointestinal (GI) peptides have been of great interest in recent years. For the first time in the history of diabetes treatment, type 2 diabetes (T2D) medications have shown benefits in cardiovascular mortality (1). One such drug class is glucagon-like peptide-1 (GLP-1) analogs, but their precise mechanisms for cardiac benefits are still being uncovered (2). Secretin, which belongs in the same family of GI peptides as GLP-1, is the first hormone discovered and with it came the concept of endocrine regulation in the 1920s (3). It is secreted during feeding and its best-established effect is induction of pancreatic exocrine secretion (4). We recently showed that secretin is not only a digestive hormone but also controls appetite and activates meal-associated brown adipose tissue thermogenesis (5, 6). Evidently, secretin has pleiotropic effects, as human secretin receptors are present in multiple organs and tissues, including the heart and kidney (7).

In the early 1980s, intravenous secretin infusion was shown to increase cardiac output and stroke volume in patients with heart failure (8) and patients with angina, but normal ventricular function (9). Since systemic resistance also fell, the secretin-induced effect was proposed to be mainly through a decrease in afterload. However, due to the substantial 20% increase in cardiac output, the authors speculated that there might also be an increase in myocardial contraction. Since then animal studies have provided support for an inotropic effect (10, 11), but it could not be proven in humans with the previously implemented method. Furthermore, several studies have also indicated that secretin has an effect on fluid homeostasis (12, 13), both centrally and through aquaporin channels in the kidneys, independent of vasopressin (14). It also increases renal blood flow (15). In earlier clinical studies, secretin was shown to have diuretic effects in humans (16–18).

Secretin’s cardiorenal effects have not been studied in humans with current, state-of-the-art methods. We recently reported the prespecified endpoints of the GUTBAT clinical trial, a randomized placebo-controlled crossover trial examining the effect of secretin on brown adipose tissue and appetite (5). In the present exploratory study, we aimed to investigate the cardiorenal effects of secretin in the participants of the GUTBAT trial (Clinical trial No.: NCT03290846).

## MATERIALS AND METHODS

### Study Subjects

Subjects (*n* = 16) who filled the inclusion criteria [body mass index (BMI) 20–26 kg/m^2^, male, age 18–65 yr, no chronic disease that would affect the outcome] and who presented healthy at the screening visit [according to their medical history, as well as when assessed by their cardiovascular status, standard 2-h oral glucose tolerance test (OGTT), routine laboratory tests, electrocardiogram (ECG), and blood pressure] were enrolled in the PET/computed tomography (CT) study. After 1 subject dropped out before the completion of the study, 15 subjects were included in the analysis [means ± standard deviation (SD) age 40.9 ± 12.5 yr, median BMI 24.0 ± 1.9 kg/m^2^]. Written informed consent was provided by all subjects. All participants were recruited between the years 2016 and 2017. The trial ended when all preplanned studies were completed (Clinical Trials Number: NCT03290846). No important harms or unintended effects were observed. The study protocol was reviewed and approved by the Ethics Committee of the Hospital District of Southwest Finland before starting the study. The study was performed according to the principles of the Declaration of Helsinki.

### Study Design

**Figure 1.**
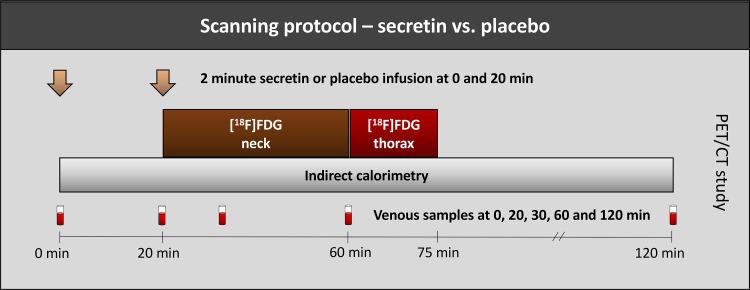
Overview of the scanning protocol. Two PET/CT scans were conducted in fasting conditions, in a single-blinded and randomized order, with a placebo (saline) and secretin (secretin pentahydrochloride 1 IU/kg *2) intervention. Arrows indicate the timing of intravenous secretin/placebo infusions. [^18^F]FDG (150 MBq) was injected at 20 min, after which PET scanning of the neck, and then the thoracic region, was initiated. Indirect calorimetry was conducted for 2 h. Timing of arterialized samples is indicated with test tube figures. Twelve-lead ECG was collected at timepoints 0, 60 and 120 min. CT, computed tomography; [^18^F]FDG, [^18^F]2-fluoro-2-deoxy-d-glucose.

The study was conducted at Turku PET Center and consisted of two separate [^18^F]2-fluoro-2-deoxy-d-glucose ([^18^F]FDG) PET scan days after ≥12 h of fasting. The scans were conducted within the interval of 2–28 days of each other (Fig. 1). Subjects were blinded to the intervention and randomized to receive placebo (saline) and secretin (secretin pentahydrochloride 1 IU/kg × 2) twice intravenously as a 2-min infusion on different days. Whole body energy expenditure (EE) was assessed with indirect calorimeter (Deltatrac II, Datex-Ohmeda) during the PET scans (19). Conventional 12-lead ECG was recorded at baseline, at 1 h, and at 2 h. Repeated arterialized venous samples were collected from the antecubital vein during the scanning days.

### Scanning Protocol

[^18^F]FDG was produced at the Turku PET Center, as described previously (20). The two PET/CT scans (GE Discovery ST System, General Electric Systems, Milwaukee, MI) were conducted according to identical scan acquisition protocols. A 2-min intravenous infusion of saline or secretin was given 20 min before the administration of 150 MBq of [^18^F]FDG. Subsequent to the radiotracer injection, a second 2-min infusion of placebo or secretin was initiated. A dynamic PET scan was started simultaneously and acquired on the neck region for 40 min [results have been reported previously (5)] and the thoracic region for 15 min (frames: 5 × 3 min) (Fig. 1). A low-dose CT was conducted before each dynamic PET scan for attenuation correction and anatomical localization.

#### Image analysis.

Image analysis was conducted with Carimas 2.8 software (Turku PET Center, Turku, Finland). An automated cardiac analysis tool was used for myocardial time-activity curves (TAC). Regional TAC data were then analyzed, taking into account radioactivity in arterialized plasma, by using fractional uptake rate (FUR) (21). Arterialized plasma radioactivity was assessed with an automatic gamma counter (Wizard 1480, Wallac, Turku, Finland). Myocardial glucose uptake (μmol/100 g/min) was calculated by multiplying FUR with plasma glucose levels and dividing by tissue density of 1.0298 g/mL (22) and a lumped constant value of 1 (23). Brown adipose tissue (BAT) and skeletal muscle glucose uptake was calculated as previously reported (5). Glucose uptake rates of these tissues are also expressed in μmol/min, by multiplying for organ mass. For the heart, the reference value of adult males (332 g) was used (22). BAT mass was measured as previously described (24) and skeletal muscle mass was estimated from age, height, weight, and waist circumference as previously described (25).

#### ECG assessments.

Twelve-lead ECGs were recorded before scanning, as well as 60 and 120 min after the first secretin or placebo dose (Fig. 1). ECG was recorded using GE Medical Systems MAC 5000 resting ECG analysis system. Heart rate, PR-, QRS, and QT intervals were automatically measured and assessed by a qualified cardiologist. Heart rate corrected QT interval (QTc) was calculated with Bazzett’s formula (26).

#### Indirect calorimetry analysis.

Analysis was started 10 min after the first secretin dose. Whole body energy expenditure (EE) and the rate at which carbohydrates (CHO) and lipids (FO) were oxidized for EE were calculated with Matlab (Version: R2011a), using the Weir equation (27) and the manufacturer’s equations (28). Whole body energy expenditure, carbohydrate oxidation, and fat oxidation by indirect calorimetry measurements are expressed in kcal/day. Protein metabolism was calculated by assuming urine nitrogen as 13 g/24 h (29).

### Renal Function Measurements

Serum samples of the first *n* = 10 scanned subjects were analyzed for a metabolic panel, including creatinine, the Nightingale Health laboratory (Helsinki, Finland), with nuclear magnetic resonance (NMR) spectroscopy (30). Glomerular filtration rate was measured with Cockcroft–Gault equation (31). All subjects voided before start of scan and after the scan. Times were recorded and urine volumes measured. Subjects received a slow saline infusion (NaCl0.9) during the scan for sampling purposes and infusion volumes were not controlled. Urine radioactivity was assessed with an automatic gamma counter at the end of the scan (Wizard 1480, Wallac, Turku, Finland). [^18^F]FDG renal clearance rate (*n* = 15) was calculated as previously described, with urine activity (FDG_urine_) divided by area under the curve (AUC) arterialized plasma radioactivity from beginning of the scan to the end (32).


Renal ClearanceFDG= FDGurineAUC0→sampling time

### Rate of Disappearance of [^18^F]FDG

The effect of secretin on whole body glucose metabolism was studied using the rate of disappearance (Rd) of glucose under placebo and secretin infusion. Rd is calculated as follows:

Rdglucose= FDGdose-FDGurineAUC0→sampling time×glucose0→end of sampling,

where FDG_dose_ is the injected [^18^F]FDG dose, FDG_urine_ is the decay-corrected quantity of [^18^F]FDG measured in the total volume of urine from [^18^F]FDG injection to the end of the PET scan, AUC is the area under the curve of [^18^F]FDG in plasma from [^18^F]FDG injection to the end of sampling, and glucose is the average glycemia from [^18^F]FDG injection to the end of sampling (33, 34).

### Statistical Analysis

Sample size calculations for the primary endpoint have been previously reported (5). Data are reported as means ± standard deviation (SD). Statistical analysis was performed with IBM SPSS Statistics (version 27). The prespecified primary and secondary endpoints of the GUTBAT Trial have been reported previously and results reported here are exploratory (5). Myocardial glucose uptake is expressed as µmol/100 g/min. Whole body carbohydrate oxidation by indirect calorimetry is expressed in kcal/day. Student’s paired *t* test was used to compare PET/CT data. Correlation was analyzed with Pearson’s correlation, unless otherwise stated. For serum creatinine analysis, R-studio was used for repeated-measures ANOVA. *P* values of <0.05 were considered as statistically significant. Randomized allocation sequences for the order of placebo and secretin interventions were generated with the randomized blocks method, with block size of 6, using SAS (v. 9.4 for Windows). The allocation sequence was generated by the Turku University statistics department that was not otherwise involved in the study. Participants were assigned to the sequence in order of enrollment, by study personnel enrolling participants into the study.

## RESULTS

**Figure 2.**
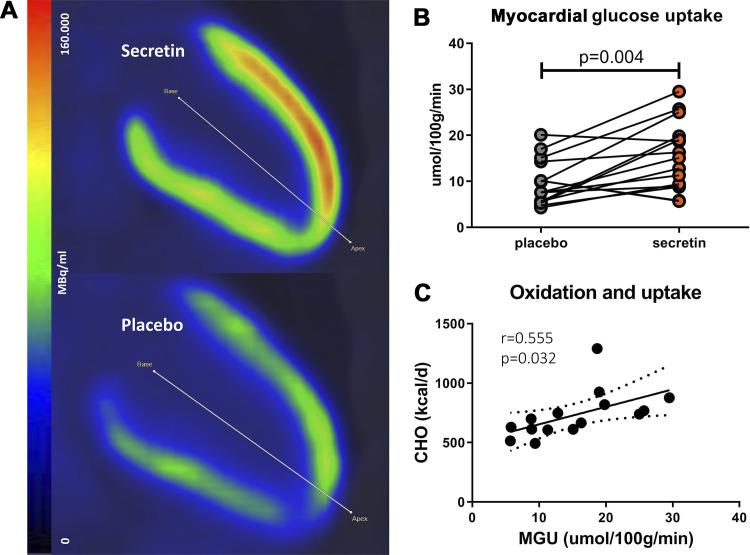
Myocardial glucose uptake results. *A*: representative [^18^F]FDG PET/CT vertical long axis images showing Ki of the heart after secretin and placebo infusions (*n* = 1). Short axis images are shown in Supplemental Fig. S1. (see https://doi.org/10.6084/m9.figshare.16912807). *B*: the effect of secretin infusion on myocardial glucose uptake compared with placebo (*n* = 15). Data were analyzed by Student’s paired *t* test. *C*: whole body carbohydrate oxidation (CHO) correlates with myocardial glucose uptake (MGU) after secretin administration (*n* = 15). Data were analyzed by Pearson correlation. A line has been drawn on the data to indicate a significant association whereas dotted curves represent confidence interval. CT, computed tomography; [^18^F]FDG, [^18^F]2-fluoro-2-deoxy-d-glucose.

Myocardial glucose uptake (GU) was significantly higher after secretin compared with placebo (15.5 ± 7.4 vs. 9.7 ± 4.9 μmol/100 g/min, *P* = 0.004; Fig. 2, *A* and *B*). Secretin-induced myocardial GU was not associated with previously reported insulin levels (5) (Spearman correlation between myocardial GU and plasma insulin at 0 min: *r* = 0.527, *P* = 0.123; 20 min: *r* = 0.275, *P* = 0.441; 60 min: *r* = 0.092, *P* = 0.800; 120 min: *r* = 0.080, *P* = 0.827). Previously reported serum insulin, glucose, and free fatty acid levels are shown in Supplemental Table S1 (see https://doi.org/10.6084/m9.figshare.16912816) (5). There was no significant difference in heart rate between the interventions at 1 h [57 ± 8 beats/min (bpm) vs. 57 ± 8 bpm, *P* = 0.92], which suggests that secretin does not have a chronotropic effect. This is supported by previous studies (8, 9). Interestingly, QTc was shortened after the two secretin infusions at 1 h compared with placebo (410.2 ± 26.1 vs. 417.0 ± 21.7 ms, *P* = 0.045). All ECG interval results are shown in Table 1.

**Table 1.** ECG intervals

Measure	Minutes	SecretinMeans ± SD	PlaceboMeans ± SD	*P* Value
Beats per minute, bpm	0	56.6 ± 7.5	55.4 ± 8.3	0.474
	60	56.8 ± 8.0	57.0 ± 7.8	0.917
	120	59.5 ± 7.8	57.8 ± 5.4	0.313
PR, mm	0	162.6 ± 24.5	158.8 ± 26.3	0.257
	60	157.4 ± 28.5	155.5 ± 24.7	0.584
	120	158.5 ± 24.7	156.8 ± 23.7	0.6
QRS, mm	0	100.5 ± 10.9	100.5 ± 10.1	1.0
	60	99.8 ± 6.6	99.8 ± 8.8	1.0
	120	100.9 ± 8.0	101.5 ± 8.5	0.524
QT, mm	0	428.0 ± 18.4	428.6 ± 18.0	0.899
	60	422.8 ± 13.2	429.5 ± 18.0	0.147
	120	421.9 ± 15.7	427.5 ± 13.9	0.122
QTc, mm	0	414.4 ± 24.1	410.2 ± 26.3	0.485
	60	410.2 ± 26.1	417.0 ± 21.7	0.045
	120	418.9 ± 24.6	418.9 ± 19.3	0.990

Values are means ± SD. ECG intervals of *n* = 15 subjects during secretin and placebo scans, measured before infusions, and 60 and 120 minutes after the first infusion. Data were analyzed by Student’s paired *t* test for each timepoint. ECG tracings of *n* = 1 subject are shown in Supplemental Fig. S2 (see https://doi.org/10.6084/m9.figshare.16912813).

To further investigate whether the increase in glucose uptake is due to increased heart work, we analyzed associations between myocardial GU and previously reported whole body energy expenditure. Whole body energy expenditure, which was 2% higher after secretin administration compared with placebo (5), was not associated with myocardial GU (*r* = −0.07, *P* = 0.79). This could indicate that the catabolic effect of secretin is not driven by heart work. However, whole body carbohydrate oxidation (CHO) was strongly associated with myocardial GU (*r* = 0.555, *P* = 0.032), which could indicate that glucose taken up by the myocardium is oxidized (Fig. 2*C*).

**Figure 3.**
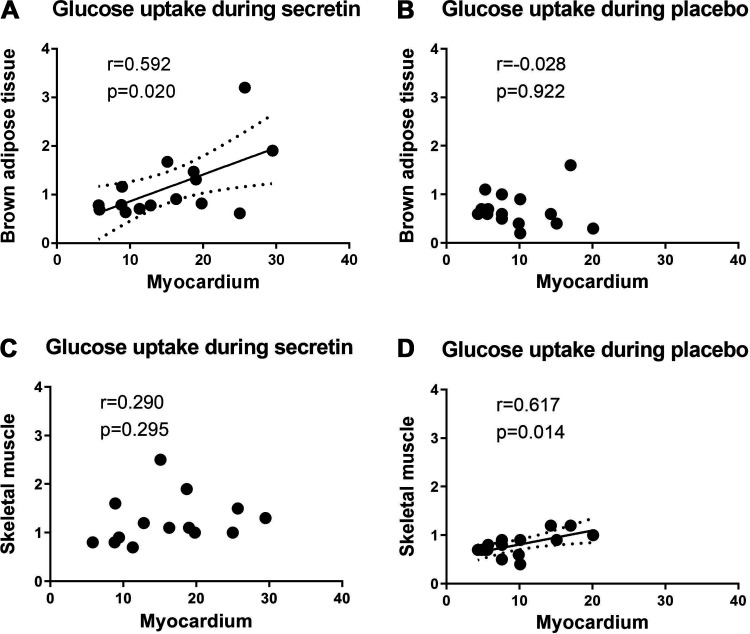
Organ glucose uptake correlations (*n* = 15). *A*: brown adipose tissue glucose uptake is strongly associated with myocardial glucose uptake after secretin infusion, while no association is seen during placebo (*B*). *C*: skeletal muscle glucose uptake is not associated with myocardial glucose uptake after secretin infusion, while an association exists during placebo (*D*). Data were analyzed by Pearson correlation. A line has been drawn on the data to indicate a significant association whereas dotted curves represent confidence interval. All units are μmol/100 g/min.

Rate of disappearance of glucose (Rd) was significantly higher after secretin administration compared with placebo (11.9 ± 2.2 vs. 10.8 ± 1.6 μmol/kg/min, *P* = 0.045), which is indicative of increased whole body GU during secretin infusion. We reported in our previous study that brown adipose tissue (BAT) and skeletal muscle glucose uptake are increased by secretin administration compared with placebo administration (5). Skeletal muscle has the largest influence on whole body glucose metabolism, due to its large mass compared with BAT and the myocardium (Supplemental Table S2; see https://doi.org/10.6084/m9.figshare.16912810). Interestingly, glucose uptake of BAT is associated with myocardial GU after the secretin infusion (*r* = 0.592, *P* = 0.020) whereas muscle GU is not (*r* = 0.270, *P* = 0.331) (Fig. 3, *A* and *C*). This could indicate that both myocardial and brown adipose tissue glucose uptakes are increased by the direct effect of secretin through secretin receptors (6), although the effect is not as pronounced in skeletal muscle. In contrast, muscle GU and myocardial GU are associated in fasting conditions (Fig. 3*D*) whereas BAT GU and myocardial GU are not (Fig. 3*B*). Fatty acids are used as an energy source in fasting conditions instead of glucose and BAT is largely inactive.

**Figure 4.**
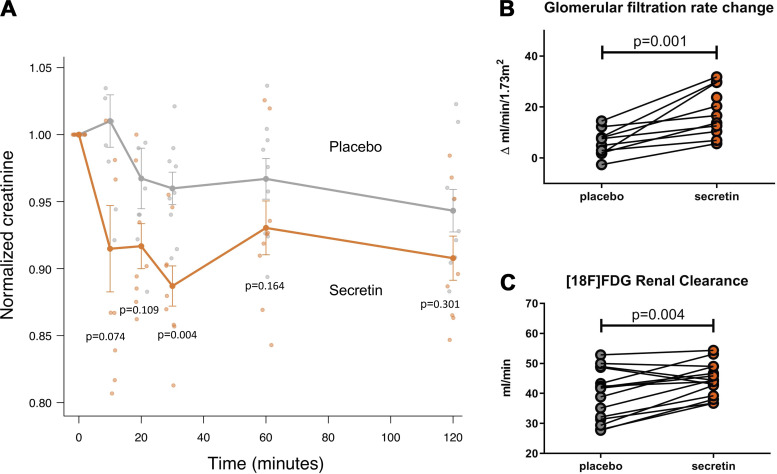
Renal function results. *A*: secretin decreases serum creatinine compared with placebo (*n* = 10). Values were normalized, dividing by the value of the first time point. Mean values and standard error are shown on graph. Each timepoint was analyzed by paired Wilcoxon signed-rank test. The secretin intervention is shown in orange whereas the placebo intervention is shown in gray. *B*: eGFR, calculated by Cockroft–Gault equation, was increased by secretin from baseline at 30 minutes, compared with placebo (*n* = 10). *C*: [^18^F]FDG renal clearance was increased after secretin (*n* = 15). *B* and *C*: data were analyzed by Student’s paired *t* test. [^18^F]FDG, [^18^F]2-fluoro-2-deoxy-d-glucose; eGFR, estimated glomerular filtration rate.

Serum creatinine was measured as part of a metabolomics panel, taken at several timepoints during the scan (Fig. 1). Serum creatinine levels decreased from baseline after secretin administration, while no such decrease was observed subsequent to placebo administration (Fig. 4*A*). Accordingly, eGFR was increased from baseline after secretin administration at 30 min compared with placebo administration (Fig. 4*B*). This is also when serum secretin levels peaked, as reported by us previously (5). [^18^F]FDG renal clearance was significantly higher after secretin administration than placebo administration (secretin vs. placebo, 44.5 ± 5.4 mL/min vs. 39.5 ± 8.5 mL/min, *P* = 0.004) (Fig. 4*C*). Urine volumes at the end of the study were not significantly different between interventions (secretin vs. placebo, 380.9 ± 138.1 vs. 338.1 ± 199.5, *P* = 0.391).

## DISCUSSION

The main findings of this study are that secretin induces an increase in myocardial glucose uptake and increases renal filtration, as indicated by the increased eGFR and clearance of [^18^F]FDG. Cardiac mortality benefits, shown by GLP-1 agonists, have sparked an interest in the cardiac effects of GI peptides, but the effects of secretin have not been previously studied in humans with modern imaging methods. Furthermore, the renal effects of any potential cardiovascular medications are of particular interest since renal failure exacerbates heart failure and vice versa. Our results in healthy, normal weight males highlight that studies on secretin in renocardiovascular pathologies are needed.

Secretin is a prandial hormone, secreted by S-cells in the duodenal epithelium. Its secretion is stimulated by the acidification of the duodenal lumen upon gastric emptying (4) and it binds to the G protein-coupled human secretin receptor (35). Secretin has an important role in the initiation of digestion, as it stimulates pancreatic exocrine secretion (4). Recently, we showed that it also has a role in postprandial thermogenesis and the termination of feeding through a gut-BAT-brain axis (6). Since cardiac output also increases postprandially (36), possibly to supply the splanchnic vasculature and facilitate nutrient distribution and digestion, we propose that secretin has a role in inducing this increase.

The cardiovascular effects of secretin were studied already 30 years ago, but possibly due to enalapril showing mortality benefits in heart failure and thus catching focus (37), and the practical pharmacological challenges of secretin being an intravenous drug with a short half-life (38), the potential of secretin as a treatment for heart failure was not further pursued. In the studies conducted by Gunnes et al, measurements were made with pulmonary artery catheterization and thermodilution technique, while ECG and femoral artery pressure were also monitored (8, 9). Secretin induced an increase in cardiac output and a drop in systemic resistance. A reduction in systemic resistance increases output, but since the increase was considerable (∼20%), the authors suggested an inotropic effect as well. Animal studies have further confirmed this. In rat cardiomyocytes, secretin receptors stimulated contraction due to accumulation of cyclic adenosine monophosphate (cAMP) in cells (10). In pigs, intracoronary secretin increased cardiac function and perfusion through nitric oxide release and β-adrenoceptors (11). In coronary endothelial cells, this was also mediated through cAMP signaling and the effects were abolished by a secretin receptor antagonist (11). cAMP production is induced by G_s_ coupled secretin receptor activation (35). Interestingly, G_q_ coupling of the receptor is also recognized and it induces intracellular calcium mobilization (35). This could also contribute to increased cardiomyocyte contraction, but to our knowledge, the mechanism has never been investigated.

In addition to the shown increase in cardiac output (8, 9), one study also showed that secretin levels are associated with normal cardiac function. Circulating gastric peptide levels were measured in patients with chronic heart failure (39). Out of the gastric peptides studied, secretin and gastrin-releasing peptide were significantly lower in patients with chronic heart failure than in controls (39). This was not the case with vasoactive intestinal peptide (VIP), gastric inhibitory peptide (GIP), insulin, or glucagon (39). This could indicate a disturbance of secretin secretion in patients with chronic heart failure. One possible explanation could be the sympathetic activation and increase in B-type natriuretic peptide (BNP) levels in patients with heart failure, which lead to increased white adipose tissue lipolysis (40). Since secretin is a powerful lipolytic agent (41), it could be downregulated between meals due to increased circulating FAs. There have not been studies addressing this question.

The heart is an omnivore, using glucose, FAs, lactate, and ketones for its metabolism, but long-chain fatty acids are the main substrate for energy metabolism (42). The rate of cardiac FA uptake is mostly determined by arterial FA concentration (43), whereas glucose uptake is regulated mainly through the recruitment of insulin-sensitive GLUT4 transporters (44). Glucose becomes the main substrate postprandially when glucose and insulin levels are high (45). It is notable, that secretin increased myocardial glucose uptake in our study, despite the concomitant increase in circulating FAs (5). Infusing FAs during a hyper insulinemic euglycemic clamp is known to decrease myocardial glucose uptake in humans (46), confirming in vivo the Randle cycle. Furthermore, fasting myocardial [^18^F]FDG uptake has been shown to correlate with increased heart work (47, 48). Taken together, our results support an inotropic effect of secretin, which was suggested in previous studies that showed an increase in cardiac output and stroke volume (8, 9).

Another interesting finding of this study was that QTc was shortened after secretin administration compared with placebo administration whereas there was no difference in heart rate. QTc interval shortens postprandially (49) and previous studies indicate that the change is not associated with insulin or glucose levels (50). In contrast, high carbohydrate uptake and high insulin levels have been tied to a postprandial increase in heart rate (51). It has been suggested that postprandial QTc shortening is associated with the signaling pathways of Ca^2+^ cycling (52). This would be in line with a known postprandial increase in inotropy, also involving Ca^2+^ cycling (52). Taken together, our results suggest that secretin could influence postprandial QTc shortening through inotropy. This warrants further studies.

SGLT2 inhibitors have sparked a new interest in the cardiorenal axis, as the drug class has shown benefits in both kidney and cardiovascular endpoints (53). Renal and heart failure exacerbate each other, and prescribed medications should ideally aim to treat both conditions simultaneously. In line with previous studies that have shown a diuretic effect of secretin (54, 55), our study also suggests a mild diuretic effect of secretin, as shown by the enhanced [^18^F]FDG renal clearance. [^18^F]FDG is filtrated into the urine, and in contrast to glucose, it is not reabsorbed by SGLT2 (56). Thus, it could reflect urine excretion. It is also of note that the increase in [^18^F]FDG clearance occurred despite higher myocardial GU during the secretin experiment. This suggests that our [^18^F]FDG clearance and glucose uptake results are, if any, under rather than overestimated.

Glomerular filtration rate was estimated using serum creatinine levels according to the Cockroft-Gault equation (31). eGFR values are dependent on not only the rate of creatinine filtration but also the rate of creatinine production by the muscle. It is thus possible that the results reflect a decrease in creatinine production. However, there is no previous literature to support this in relation to secretin, and our [^18^F]FDG renal clearance rate results support an increase in glomerular filtration. Interestingly, creatinine clearance rate has previously been shown to associate with brown adipose tissue activation in humans (57). The authors proposed that this could reflect an increase in creatine phosphate turnover, as eGFR also increases after muscular exercise (58). Beige adipose tissue thermogenesis is enhanced by a creatine-driven substrate cycle (59), one that has also been shown to have a role in diet-induced thermogenesis in mice (60). The study showed that the genetic depletion of adipose creatine metabolism drives diet-induced obesity (60). Since we previously showed that secretin activates brown adipose tissue (6), the increase in eGFR could also reflect BAT activation. Furthermore, brown adipose tissue glucose uptake was associated with myocardial glucose uptake after secretin, which indicates that the effects are linked. This is an interesting finding since brown adipose tissue activity has recently been shown to associate with cardiovascular health, especially in patients with obesity (61).

Strengths of the present study are the use of state-of-the-art techniques to measure myocardial substrate uptake rates in vivo and the randomized placebo-controlled crossover protocol used. Our study also has limitations. First, the sample size, even though sufficient for the primary endpoints of this clinical trial, was relatively small. Infusion volumes of saline were not controlled since urine volume was not a prespecified endpoint in this study. These factors may have precluded us from finding a significant change in urine volume excretion induced by secretin. Our subjects were all healthy males and further studies are needed to determine whether our results are applicable to a wider population.

In conclusion, our study supports the previous hypothesis that secretin has an inotropic effect in humans. This is the first study to demonstrate that secretin may directly impact myocardial glucose metabolism, despite concomitant increase in circulating FA levels. We also showed for the first time that secretin induces QTc shortening, which is known to occur postprandially. Our results also indicate that secretin increases renal filtration in humans, suggesting that the hormone has an influence on the cardiorenal axis. Based on the present findings, we believe that larger studies are warranted to investigate whether secretin may have a place in the future treatment of heart failure.

## SUPPLEMENTAL DATA

10.6084/m9.figshare.16912816 Supplemental Table S1: https://doi.org/10.6084/m9.figshare.16912816

10.6084/m9.figshare.16912810 Supplemental Table S2: https://doi.org/10.6084/m9.figshare.16912810

10.6084/m9.figshare.16912807 Supplemental Fig. S1: https://doi.org/10.6084/m9.figshare.16912807

10.6084/m9.figshare.16912813 Supplemental Fig. S2: https://doi.org/10.6084/m9.figshare.16912813.

## GRANTS

The study was conducted within the Center of Excellence into Cardiovascular and Metabolic Diseases supported by the Academy of Finland (307402), University of Turku, Åbo Akademi University. The study was also funded by the Instrumentarium Science Foundation (190014) (to S.L.), The Paulo Foundation (to S.L.), Turku University Hospital Foundation (to S.L.), and The Finnish Medical Foundation (2985) (to S.L.).

## DISCLOSURES

M.K. is an inventor on a patent application from the Technical University of Munich (Publication No. WO/2017/20285; International Application No. PCT/EP2017/062420) addressing the role of secretin receptor agonists and modulators in the regulation of energy homeostasis. This patent is based on the initial discovery that meal-induced secretin inhibits food intake, and this anorexigenic action of secretin depends on the activation of brown fat (6). None of the other authors has any conflicts of interest, financial or otherwise, to disclose.

## AUTHOR CONTRIBUTIONS

S.L., K.S., L.N., M.K., K.A.V., and P.N., conceived and designed research; S.L., M.L., and L.S. performed experiments; S.L., E.R., T.-M.P., R.K., M.U.-D., and M-J.H. analyzed data; S.L. and E.R. interpreted results of experiments; S.L. and R.K. prepared figures; S.L. and E.R. drafted manuscript; S.L., E.R., K.S., R.K., and M.K., edited and revised manuscript; S.L., E.R., M.L., L.S., K.S., T.-M.P., R.K., M.U.-D. M.-J.H., O.E., A.K.K., L.N., M.K., K.A.V., and P.N. approved final version of manuscript.

## References

[1] Cosentino F, Grant PJ, Aboyans V, Bailey CJ, Ceriello A, Delgado V, Federici M, Filippatos G, Grobbee DE, Hansen TB, Huikuri HV, Johansson I, Jüni P, Lettino M, Marx N, Mellbin LG, Östgren CJ, Rocca B, Roffi M, Sattar N, Seferović PM, Sousa-Uva M, Valensi P, Wheeler DC; ESC Scientific Document Group. 2019 ESC Guidelines on diabetes, pre-diabetes, and cardiovascular diseases developed in collaboration with the EASD. Eur Heart J 41: 255–323, 2020. doi:10.1093/eurheartj/ehz486. 31497854

[2] Nauck MA, Meier JJ, Cavender MA, El Aziz MA, Drucker DJ. Cardiovascular actions and clinical outcomes with glucagon-like peptide-1 receptor agonists and dipeptidyl peptidase-4 inhibitors. Circulation 136: 849–870, 2017. doi:10.1161/CIRCULATIONAHA.117.028136. 28847797

[3] Bayliss WM, Starling EH. The mechanism of pancreatic secretion. J Physiol 28: 325–353, 1902. doi:10.1113/jphysiol.1902.sp000920. 16992627 PMC1540572

[4] Kim MS, Lee KY, Chey WY. Plasma secretin concentrations in fasting and postprandial states in dog. Am J Physiol Endocrinol Physiol 5: E539–E544, 1979. doi:10.1152/ajpendo.1979.236.5.e539. 35981

[5] Laurila S, Sun L, Lahesmaa M, Schnabl K, Laitinen K, Klén R, Li Y, Balaz M, Wolfrum C, Steiger K, Niemi T, Taittonen M, U-Din M, Välikangas T, Elo LL, Eskola O, Kirjavainen AK, Nummenmaa L, Virtanen KA, Klingenspor M, Nuutila P. Secretin activates brown fat and induces satiation. Nat Metab 3: 798–809, 2021. doi:10.1038/s42255-021-00409-4. 34158656

[6] Li Y, Schnabl K, Gabler S-MM, Willershäuser M, Reber J, Karlas A, Laurila S, Lahesmaa M, U Din M, Bast-Habersbrunner A, Virtanen KA, Fromme T, Bolze F, O'Farrell LS, Alsina-Fernandez J, Coskun T, Ntziachristos V, Nuutila P, Klingenspor M. Secretin-Activated brown fat mediates prandial thermogenesis to induce satiation. Cell 175: 1561–1574.e12, 2018. doi:10.1016/j.cell.2018.10.016. 30449620

[7] Chu JYS, Yung WH, Chow BKC. Secretin: a pleiotrophic hormone. Ann N Y Acad Sci 1070: 27–50, 2006. doi:10.1196/annals.1317.013. 16888148

[8] Gunnes P, Rasmussen K. Haemodynamic effects of pharmacological doses of secretin in patients with impaired left ventricular function. Eur Heart J 7: 146–149, 1986. doi:10.1093/oxfordjournals.eurheartj.a062037. 3699051

[9] Gunnes P, Waldum HL, Rasmussen K, Ostensen H, Burhol PG. Cardiovascular effects of secretin infusion in man. Scand J Clin Lab Invest 43: 637–642, 1983. 6658378

[10] Bell D, McDermott BJ. Secretin and vasoactive intestinal peptide are potent stimulants of cellular contraction and accumulation of cyclic AMP in rat ventricular cardiomyocytes. J Cardiovasc Pharmacol 23: 959–969, 1994. doi:10.1097/00005344-199406000-00015. 7523789

[11] Grossini E, Molinari C, Morsanuto V, Mary DASG, Vacca G. Intracoronary secretin increases cardiac perfusion and function in anaesthetized pigs through pathways involving β-adrenoceptors and nitric oxide. Exp Physiol 98: 973–987, 2013. doi:10.1113/expphysiol.2012.070607. 23243148

[12] Chu JYS, Cheng CYY, Lee VHY, Chan Y, Chow BKC. Secretin and body fluid homeostasis. Kidney Int 79: 280–287, 2011. doi:10.1038/ki.2010.397. 20944548

[13] Chu JYS, Lee LTO, Lai CH, Vaudry H, Chan YS, Yung WH, Chow BKC. Secretin as a neurohypophysial factor regulating body water homeostasis. Proc Natl Acad Sci USA 106: 15961–15966, 2009. doi:10.1073/pnas.0903695106. 19805236 PMC2747226

[14] Chu JYS, Chung SCK, Lam AKM, Tam S, Chung S, Chow BKC. Phenotypes developed in secretin receptor-null mice indicated a role for secretin in regulating renal water reabsorption. Mol Cell Biol 27: 2499–2511, 2007. doi:10.1128/MCB.01088-06. 17283064 PMC1899889

[15] Waldum HL, Sundsfjord JA, Aanstad U, Burhol PG. The effect of secretin on renal haemodynamics in man. Scand J Clin Lab Invest 40: 475–478, 1980. doi:10.3109/00365518009101870. 7444349

[16] Baron DN, Newman F, Warrick A. The effects of secretin on urinary volume and electrolytes in normal subjects and patients with chronic pancreatic disease. Experientia 14: 30–32, 1958. doi:10.1007/BF02159660.

[17] Viteri AL, Poppell JW, Lasater JM, Dyck WP. Renal response to secretin. J Appl Physiol 38: 661–664, 1975. doi:10.1152/jappl.1975.38.4.661. 237865

[18] Londong W, Londong V, Mühlbauer R, König A. Pharmacological effects of secretin and somatostatin on gastric and renal function in man. Scand J Gastroenterol Suppl 139: 25–31, 1987. doi:10.3109/00365528709089771. 2893448

[19] Orava J, Nuutila P, Lidell ME, Oikonen V, Noponen T, Viljanen T, Scheinin M, Taittonen M, Niemi T, Enerbäck S, Virtanen KA. Different metabolic responses of human brown adipose tissue to activation by cold and insulin. Cell Metab 14: 272–279, 2011. doi:10.1016/j.cmet.2011.06.012. 21803297

[20] Hamacher K, Coenen HH, Stöcklin G. Efficient stereospecific synthesis of no-carrier-added 2-[18F]-fluoro-2-deoxy-D-glucose using aminopolyether supported nucleophilic substitution. J Nucl Med 27: 235–238, 1986. 3712040

[21] Thie JA. Clarification of a fractional uptake concept. J Nucl Med 36: 711–712, 1995. 7699475

[22] Report of the task group on reference man ICRP Publication 23. Ann ICRP 4 : III–III. doi:10.1016/0146-6453(80)90047-0. 20863799

[23] Bøtker HE, Böttcher M, Schmitz O, Gee A, Hansen SB, Cold GE, Nielsen TT, Gjedde A. Glucose uptake and lumped constant variability in normal human hearts determined with [^18^F]fluorodeoxyglucose. J Nucl Cardiol 4: 125–132, 1997. doi:10.1016/s1071-3581(97)90061-1. 9115064

[24] U Din M, Saari T, Raiko J, Kudomi N, Maurer SF, Lahesmaa M, Fromme T, Amri EZ, Klingenspor M, Solin O, Nuutila P, Virtanen KA. Postprandial oxidative metabolism of human brown fat indicates thermogenesis. Cell Metab 28: 207–216.e3, 2018. doi:10.1016/j.cmet.2018.05.020. 29909972

[25] Heymsfield SB, Stanley A, Pietrobelli A, Heo M. Simple skeletal muscle mass estimation formulas: what we can learn from them. Front Endocrinol (Lausanne) 11: 31, 2020. doi:10.3389/FENDO.2020.00031. 32117059 PMC7012897

[26] Bazzett H. An analysis of the time-relations of electrocardiograms. Heart 7: 353–337, 1920. doi:10.1016/j.vascn.2005.02.005. 15886026

[27] Weir JBDE. New methods for calculating metabolic rate with special reference to protein metabolism. J Physiol 109: 1–9, 1949. doi:10.1113/jphysiol.1949.sp004363. 15394301 PMC1392602

[28] Meriläinen PT. Metabolic monitor. Int J Clin Monit Comput 4: 167–177, 1987. doi:10.1007/BF02915904. 3116131

[29] U-Din M, Raiko J, Saari T, Kudomi N, Tolvanen T, Oikonen V, Teuho J, Sipilä HT, Savisto N, Parkkola R, Nuutila P, Virtanen KA. Human brown adipose tissue [15O]O2 PET imaging in the presence and absence of cold stimulus. Eur J Nucl Med Mol Imaging 43: 1878–1886, 2016 doi:10.1007/s00259-016-3364-y.[26993316] 26993316 PMC4969352

[30] Soininen P, Kangas AJ, Würtz P, Tukiainen T, Tynkkynen T, Laatikainen R, Järvelin M-R, Kähönen M, Lehtimäki T, Viikari J, Raitakari OT, Savolainen MJ, Ala-Korpela M. High-throughput serum NMR metabonomics for cost-effective holistic studies on systemic metabolism. Analyst 134: 1781–1785, 2009. doi:10.1039/b910205a. 19684899

[31] Cockcroft DW, Gault MH. Prediction of creatinine clearance from serum creatinine. Nephron 16: 31–41, 1976. doi:10.1159/000180580. 1244564

[32] Latva-Rasku A, Honka MJ, Kullberg J, Mononen N, Lehtimäki T, Saltevo J, Kirjavainen AK, Saunavaara V, Iozzo P, Johansson L, Oscarsson J, Hannukainen JC, Nuutila P. The SGLT2 inhibitor dapagliflozin reduces liver fat but does not affect tissue insulin sensitivity: a randomized, double-blind, placebo-controlled study with 8-week treatment in type 2 diabetes patients. Diabetes Care 42: 931–937, 2019. doi:10.2337/dc18-1569. 30885955

[33] Iozzo P, Gastaldelli A, Järvisalo MJ, Kiss J, Borra R, Buzzigoli E, Viljanen A, Naum G, Viljanen T, Oikonen V, Knuuti J, Savunen T, Salvadori PA, Ferrannini E, Nuutila P. 18F-FDG assessment of glucose disposal and production rates during fasting and insulin stimulation: a validation study. J Nucl Med 47: 1016–1022, 2006. 16741312

[34] Rebelos E, Immonen H, Bucci M, Hannukainen JC, Nummenmaa L, Honka M-J, Soinio M, Salminen P, Ferrannini E, Iozzo P, Nuutila P. Brain glucose uptake is associated with endogenous glucose production in obese patients before and after bariatric surgery and predicts metabolic outcome at follow-up. Diabetes Obes Metab 21: 218–226, 2019. doi:10.1111/dom.13501. 30098134 PMC6586041

[35] Garcia GL, Dong M, Miller LJ. Differential determinants for coupling of distinct G proteins with the class B secretin receptor. Am J Physiol Cell Physiol 302: C1202–C1212, 2012. doi:10.1152/ajpcell.00273.2011. 22277758 PMC3330731

[36] Waaler BA, Eriksen M, Toska K. The effect of meal size on postprandial increase in cardiac output. Acta Physiol Scand 142: 33–39, 1991. doi:10.1111/j.1748-1716.1991.tb09125.x. 1877363

[37] CONSENSUS Trial Study Group. Effects of enalapril on mortality in severe congestive heart failure. N Engl J Med 316: 1429–1435, 1987. doi:10.1056/nejm198706043162301. 2883575

[38] Laurila S, Rebelos E, Honka M-J, Nuutila P. Pleiotropic effects of secretin: a potential drug candidate in the treatment of obesity? Front Endocrinol (Lausanne) 12: 737686, 2021. doi:10.3389/fendo.2021.737686. 34671320 PMC8522834

[39] Nicholls DP, Riley M, Elborn JS, Stanford CF, Shaw C, McKillop JM, Buchanan KD. Regulatory peptides in the plasma of patients with chronic cardiac failure at rest and during exercise. Eur Heart J 13: 1399–1404, 1992. doi:10.1093/oxfordjournals.eurheartj.a060073. 1396815

[40] Kintscher U, Foryst-Ludwig A, Haemmerle G, Zechner R. The role of adipose triglyceride lipase and cytosolic lipolysis in cardiac function and heart failure. Cell Rep Med 1: 100001, 2020. doi:10.1016/j.xcrm.2020.100001. 33205054 PMC7659492

[41] Sekar R, Chow BKC. Lipolytic actions of secretin in mouse adipocytes. J Lipid Res 55: 190–200, 2014. doi:10.1194/jlr.M038042. 24273196 PMC3886658

[42] Depre C, Vanoverschelde J-L, Taegtmeyer H. Glucose for the heart? Circulation 99: 578–588, 1999. PMC] doi:10.1161/01.cir.99.4.578. 9927407

[43] An D, Rodrigues B. Role of changes in cardiac metabolism in development of diabetic cardiomyopathy. Am J Physiol Heart Circ Physiol 291: H1489–H506, 2006. doi:10.1152/ajpheart.00278.2006. 16751293

[44] Lopaschuk GD, Stanley WC. Glucose metabolism in the ischemic heart. Circulation 95: 313–315, 1997. doi:10.1161/01.CIR.95.2.313. 9008441

[45] Taegtmeyer H. Energy metabolism of the heart: from basic concepts to clinical applications. Curr Probl Cardiol 19: 59–113, 1994. doi:10.1016/0146-2806(94)90008-6. 8174388

[46] Nuutila P, Koivisto VA, Knuuti J, Ruotsalainen U, Teräs M, Haaparanta M, Bergman J, Solin O, Voipio-Pulkki L-M, Wegelius U, Yki-Järvinen H. Glucose-free fatty acid cycle operates in human heart and skeletal muscle in vivo. J Clin Invest 89: 1767–1744, 1992. doi:10.1172/JCI115780. 1601987 PMC295871

[47] Duchenne J, Turco A, Ünlü S, Pagourelias ED, Vunckx K, Degtiarova G, Bézy S, Cvijic M, Nuyts J, Claus P, Rega F, Gheysens O, Voigt J-U. Left ventricular remodeling results in homogenization of myocardial work distribution. Circ Arrhythm Electrophysiol 12: e007224, 2019. doi:10.1161/CIRCEP.118.007224. 31023060

[48] Masci PG, Marinelli M, Piacenti M, Lorenzoni V, Positano V, Lombardi M, L’Abbate A, Neglia D. Myocardial structural, perfusion, and metabolic correlates of left bundle branch block mechanical derangement in patients with dilated cardiomyopathy: a tagged cardiac magnetic resonance and positron emission tomography study. Circ Cardiovasc Imaging 3: 482–490, 2010. doi:10.1161/CIRCIMAGING.109.934638. 20463209

[49] Taubel J, Wong AH, Naseem A, Ferber G, Camm AJ. Shortening of the QT interval after food can be used to demonstrate assay sensitivity in thorough QT studies. J Clin Pharmacol 52: 1558–1565, 2012. doi:10.1177/0091270011419851. 22067197

[50] Taubel J, Lorch U, Ferber G, Singh J, Batchvarov VN, Savelieva I, Camm AJ. Insulin at normal physiological levels does not prolong QT_c_ interval in thorough QT studies performed in healthy volunteers. Br J Clin Pharmacol 75: 392–403, 2013. doi:10.1111/j.1365-2125.2012.04376.x. 22775199 PMC3579254

[51] Scott EM, Greenwood JP, Vacca G, Stoker JB, Gilbey SG, Mary DASG. Carbohydrate ingestion, with transient endogenous insulinaemia, produces both sympathetic activation and vasodilatation in normal humans. Clin Sci (Lond) 102: 523–529, 2002. 11980571

[52] Täubel J, Ferber G, Van Langenhoven L, del Bianco T, Fernandes S, Djumanov D, Kanters JK, Graff C, Camm AJ. The cardiovascular effects of a meal: J-T_peak_ and T_peak_-T_end_ assessment and further insights into the physiological effects. J Clin Pharmacol 59: 799–810, 2019. doi:10.1002/jcph.1374. 30633366 PMC6590239

[53] van Bommel EJM, Muskiet MHA, van Baar MJB, Tonneijck L, Smits MM, Emanuel AL, Bozovic A, Danser AHJ, Geurts F, Hoorn EJ, Touw DJ, Larsen EL, Poulsen HE, Kramer MHH, Nieuwdorp M, Joles JA, van Raalte DH. The renal hemodynamic effects of the SGLT2 inhibitor dapagliflozin are caused by post-glomerular vasodilatation rather than pre-glomerular vasoconstriction in metformin-treated patients with type 2 diabetes in the randomized, double-blind RED trial. Kidney Int 97: 202–212, 2020. doi:10.1016/j.kint.2019.09.013. 31791665

[54] Barbezat GO, Isenberg JI, Grossman MI. Diuretic action of secretin in dog. Proc Soc Exp Biol Med 139: 211–215, 1972. doi:10.3181/00379727-139-36111. 5007464

[55] Owen SE, Ivy AC. The diuretic action of secretin preparations. Am J Physiol Content 97: 276–281, 1931. doi:10.1152/ajplegacy.1931.97.2.276.

[56] Oikonen V. Quantification of metabolic rate of glucose uptake with [^18^F]FDG (Online). [Accessed Oct 12, 2021] http://www.turkupetcentre.net/petanalysis/analysis_18f-fdg.html.

[57] Gerngroß C, Schretter J, Klingenspor M, Schwaiger M, Fromme T. Active brown fat during ^18^F-FDG PET/CT imaging defines a patient group with characteristic traits and an increased probability of brown fat redetection. J Nucl Med 58: 1104–1110, 2017. doi:10.2967/jnumed.116.183988. 28104743

[58] Refsum HE, Strömme SB. Urea and creatinine production and excretion in urine during and after prolonged heavy exercise. Scand J Clin Lab Invest 33: 247–254, 1974. doi:10.1080/00365517409082493. 4855337

[59] Kazak L, Chouchani ET, Jedrychowski MP, Erickson BK, Shinoda K, Cohen P, Vetrivelan R, Lu GZ, Laznik-Bogoslavski D, Hasenfuss SC, Kajimura S, Gygi SP, Spiegelman BM. A creatine-driven substrate cycle enhances energy expenditure and thermogenesis in beige fat. Cell 163: 643–655, 2015. doi:10.1016/j.cell.2015.09.035. 26496606 PMC4656041

[60] Kazak L, Chouchani ET, Lu GZ, Jedrychowski MP, Bare CJ, Mina AI, Kumari M, Zhang S, Vuckovic I, Laznik-Bogoslavski D, Dzeja P, Banks AS, Rosen ED, Spiegelman BM. Genetic depletion of adipocyte creatine metabolism inhibits diet-induced thermogenesis and drives obesity. Cell Metab 26: 660–671.e3, 2017. doi:10.1016/j.cmet.2017.08.009. 28844881 PMC5629120

[61] Becher T, Palanisamy S, Kramer DJ, Eljalby M, Marx SJ, Wibmer AG, Butler SD, Jiang CS, Vaughan R, Schöder H, Mark A, Cohen P. Brown adipose tissue is associated with cardiometabolic health. Nat Med 27: 58–65, 2021. doi:10.1038/s41591-020-1126-7. 33398160 PMC8461455

